# Supplementary figures and images for: A native chemical chaperone in the human eye lens
Source: eLife. 2022 Jun 20;11:e76923. doi: 10.7554/eLife.76923 (PMC9246369; doi:10.7554/eLife.76923)

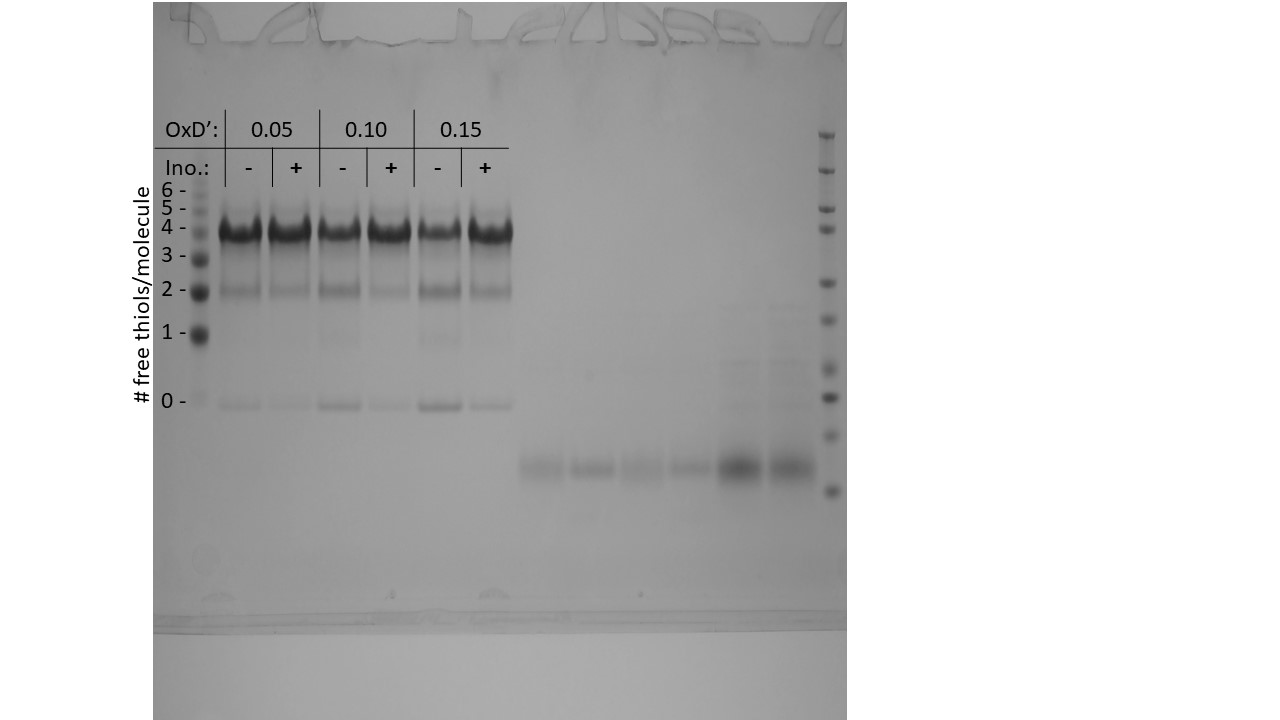

Supplement: Figure 6—figure supplement 1—source data 1. [file elife-76923-fig6-figsupp1-data1.zip › Figure 6 - figure supplement 1 - source data/Figure 6 - figure supplement 1 - source data 1 labeled gel.jpg]

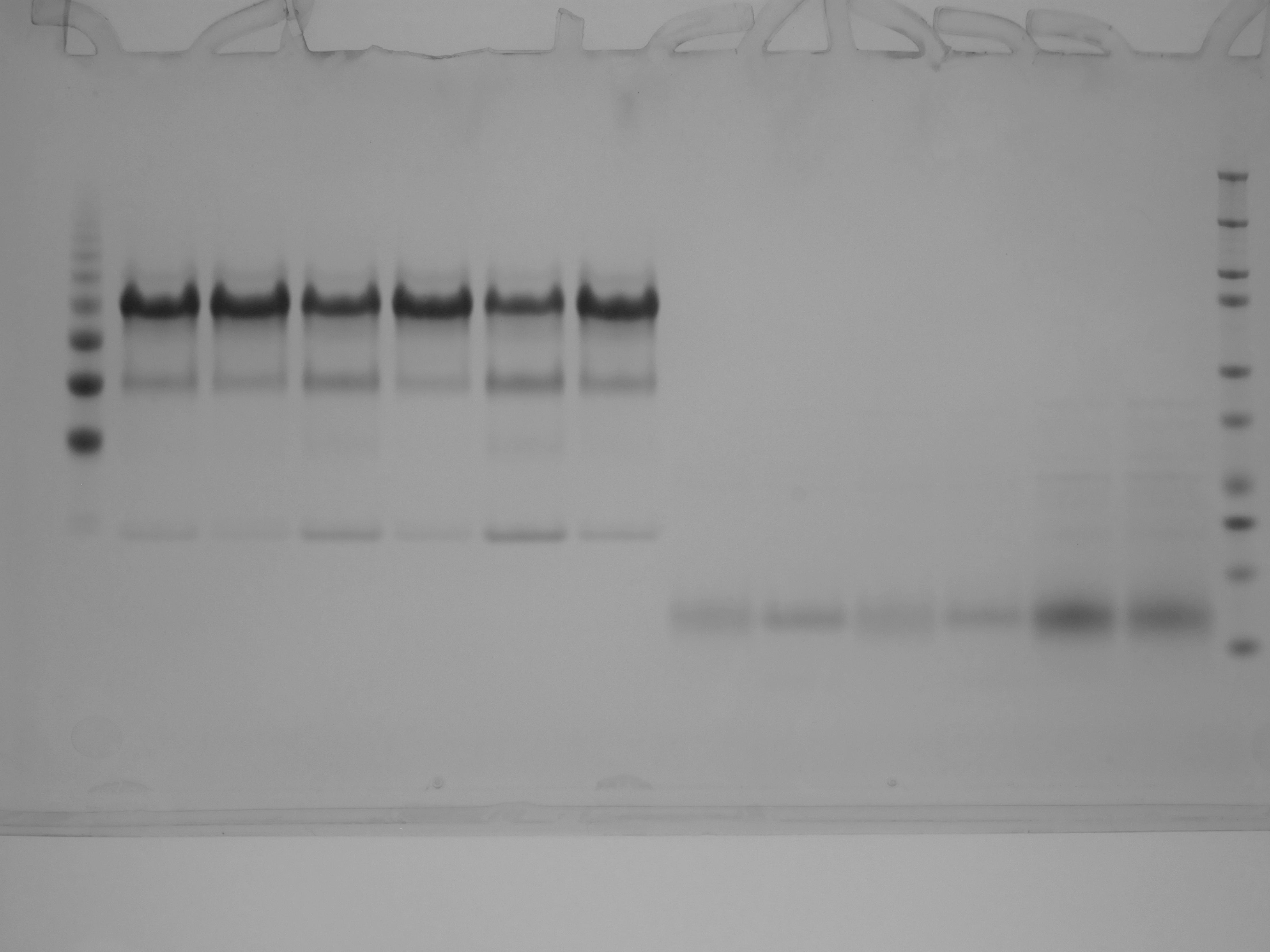

Supplement: Figure 6—figure supplement 1—source data 1. [file elife-76923-fig6-figsupp1-data1.zip › Figure 6 - figure supplement 1 - source data/Figure 6 - figure supplement 1 - source data 1.JPG]

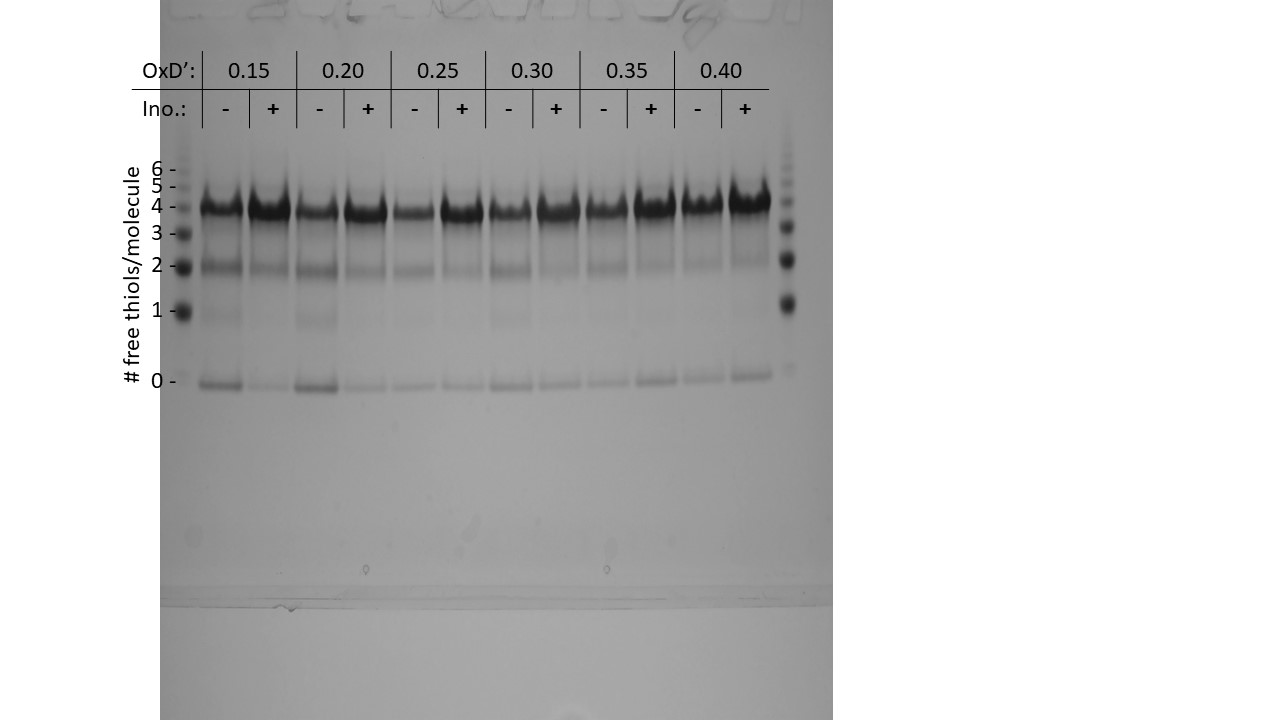

Supplement: Figure 6—figure supplement 1—source data 1. [file elife-76923-fig6-figsupp1-data1.zip › Figure 6 - figure supplement 1 - source data/Figure 6 - figure supplement 1 - source data 2 labeled gel.jpg]

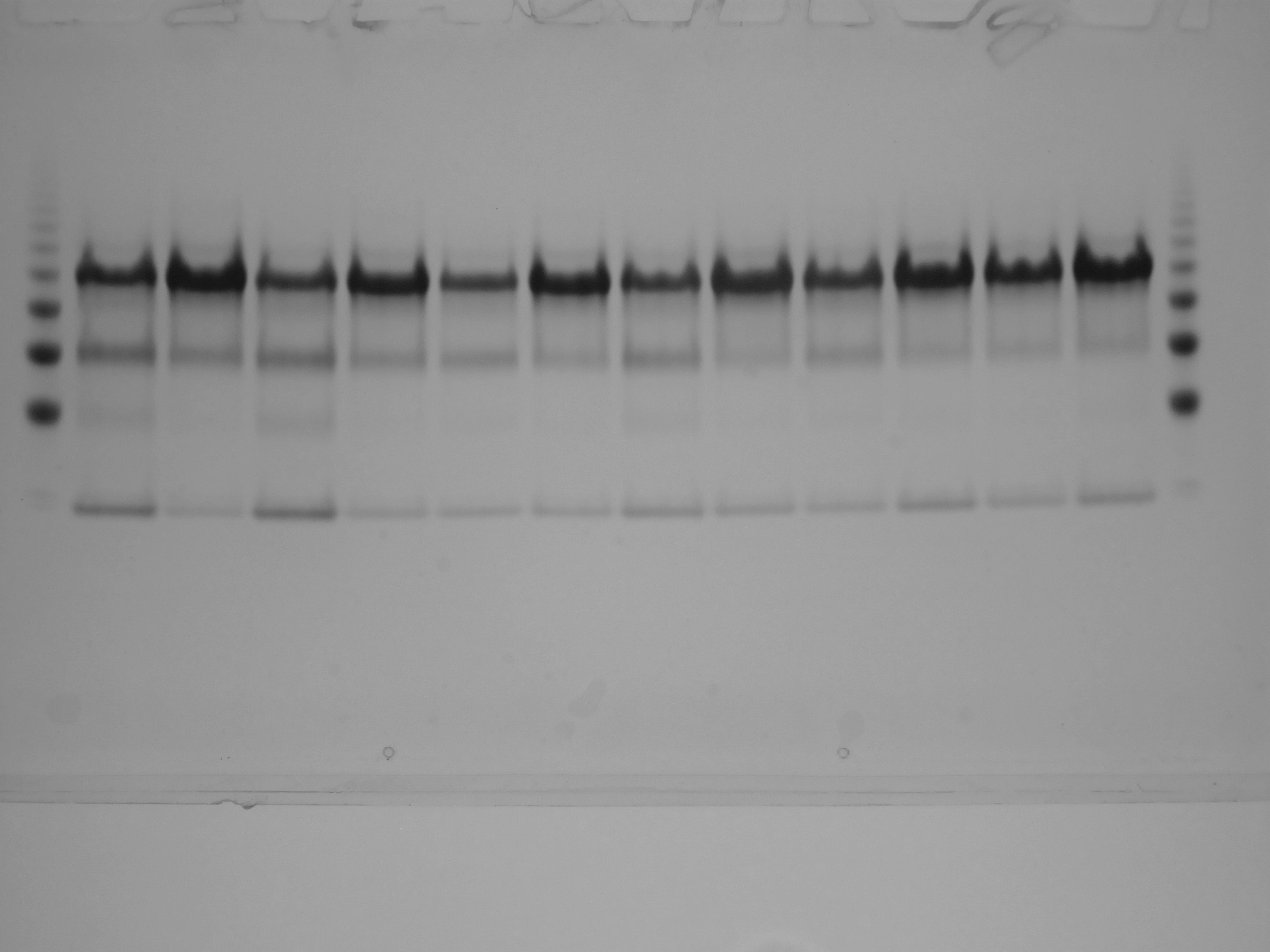

Supplement: Figure 6—figure supplement 1—source data 1. [file elife-76923-fig6-figsupp1-data1.zip › Figure 6 - figure supplement 1 - source data/Figure 6 - figure supplement 1 - source data 2.JPG]
