# Supplementary material for: A native chemical chaperone in the human eye lens
Source: eLife. 2022 Jun 20;11:e76923. doi: 10.7554/eLife.76923 (PMC9246369; doi:10.7554/eLife.76923)
Supplement: Transparent reporting form [file elife-76923-transrepform1.docx]

***eLife’s* transparent reporting form**

We encourage authors to provide detailed information *within their submission* to facilitate the interpretation and replication of experiments. Authors can upload supporting documentation to indicate the use of appropriate reporting guidelines for health-related research (see [EQUATOR Network](http://www.equator-network.org/%20)), life science research (see the [BioSharing Information Resource](https://biosharing.org/)), or the [ARRIVE guidelines](http://www.plosbiology.org/article/info:doi/10.1371/journal.pbio.1000412) for reporting work involving animal research. Where applicable, authors should refer to any relevant reporting standards documents in this form.

If you have any questions, please consult our Journal Policies and/or contact us: [editorial@elifesciences.org](mailto:editorial@elifesciences.org).

**Sample-size estimation**

- You should state whether an appropriate sample size was computed when the study was being designed
- You should state the statistical method of sample size computation and any required assumptions
- If no explicit power analysis was used, you should describe how you decided what sample (replicate) size (number) to use

Please outline where this information can be found within the submission (e.g., sections or figure legends), or explain why this information doesn’t apply to your submission:

Since this is an in-vitro molecular biochemical/biophysical investigation, sample sizes were not estimated in advance.

**Replicates**

- You should report how often each experiment was performed
- You should include a definition of biological versus technical replication
- The data obtained should be provided and sufficient information should be provided to indicate the number of independent biological and/or technical replicates
- If you encountered any outliers, you should describe how these were handled
- Criteria for exclusion/inclusion of data should be clearly stated
- High-throughput sequence data should be uploaded before submission, with a private link for reviewers provided (these are available from both GEO and ArrayExpress)

Please outline where this information can be found within the submission (e.g., sections or figure legends), or explain why this information doesn’t apply to your submission:

The relevant descriptions are included in the Figure legends. Error bars showing standard error of the mean are included in the Figures where needed. Dose-response series were used in place of replicates in certain cases where the trends were evident by inspection. EM images were only excluded if visible tears were present in the grid coating. Biological replicates were defined as experiments set up and conducted separately on different days; technical replicates were set up and measured in parallel or immediately in sequence.

**Statistical reporting**

- Statistical analysis methods should be described and justified
- Raw data should be presented in figures whenever informative to do so (typically when N per group is less than 10)
- For each experiment, you should identify the statistical tests used, exact values of N, definitions of center, methods of multiple test correction, and dispersion and precision measures (e.g., mean, median, SD, SEM, confidence intervals; and, for the major substantive results, a measure of effect size (e.g., Pearson's r, Cohen's d)
- Report exact p-values wherever possible alongside the summary statistics and 95% confidence intervals. These should be reported for all key questions and not only when the p-value is less than 0.05.

Please outline where this information can be found within the submission (e.g., sections or figure legends), or explain why this information doesn’t apply to your submission:

Statistical reporting can be found in the relevant Figures and Figure legends. Data points in Figure 1B; Figure 4B,C,E,F; and Figure 7 are reported as mean +/- S.E.M. Most trends were evident without statistical analysis. However, in the case of comparing TEM size distributions (Figure 4D), the two-sided Kolmogorov-Smirnov test was employed and p-values reported as described in Results on page 6 of the main text. This test is suitable for establishing the likelihood that two datasets came from distributions of the same (normalized) shape, regardless of the shape. The numerical values are uploaded as a supplementary table (Figure 4—supplement1), and the raw images themselves have been posted as a dataset on the Dataverse repository.

(For large datasets, or papers with a very large number of statistical tests, you may upload a single table file with tests, Ns, etc., with reference to sections in the manuscript.)

**Group allocation**

- Indicate how samples were allocated into experimental groups (in the case of clinical studies, please specify allocation to treatment method); if randomization was used, please also state if restricted randomization was applied
- Indicate if masking was used during group allocation, data collection and/or data analysis

Please outline where this information can be found within the submission (e.g., sections or figure legends), or explain why this information doesn’t apply to your submission:

The only method that required some subjective interpretation and cataloging was TEM morphometry. For this method, a double-blind was set up as described in Results on page 6 of the main text of the preprint. Samples were deidentified and imaged in randomized order; images were deidentified and randomized prior to image analysis.

**Additional data files (“source data”)**

- We encourage you to upload relevant additional data files, such as numerical data that are represented as a graph in a figure, or as a summary table
- Where provided, these should be in the most useful format, and they can be uploaded as “Source data” files linked to a main figure or table
- Include model definition files including the full list of parameters used
- Include code used for data analysis (e.g., R, MatLab)
- Avoid stating that data files are “available upon request”

Please indicate the figures or tables for which source data files have been provided:

Numerical data and fits used to generate the plots and fits of Figure 1B,C,D,E have been uploaded as source data files. Raw numerical measurements behind the graphs presented in Figure 4 are included as a supplementary table (Figure 4—supplement1). All raw negative-stain TEM images and instrument data files have been uploaded to the Dataverse repository and made publicly available at https://doi.org/10.7910/DVN/BVRS9M. Full unedited gels used to generate Figure 6 – figure supplement 1 were uploaded as source data. The numerical data and fits used to generate Figure 6D were uploaded as a source data file. All other data are contained in the manuscript itself.
